# Insomnia in tension-type headache: a population-based study

**DOI:** 10.1186/s10194-017-0805-3

**Published:** 2017-09-12

**Authors:** Jiyoung Kim, Soo-Jin Cho, Won-Joo Kim, Kwang Ik Yang, Chang-Ho Yun, Min Kyung Chu

**Affiliations:** 1 Department of Neurology, Bio Medical Research Institute, Pusan National University Hospital, Pusan National University School of Medicine, Busan, Korea; 2 Department of Neurology, Dongtan Sacred Heart Hospital, Hallym University College of Medicine, Hwaseong, Korea; 3 Department of Neurology, Gangnam Severance Hospital, Yonsei University, College of Medicine, Seoul, Korea; 4 Department of Neurology, Soonchunhyang University College of Medicine, Cheonan Hospital, Cheonan, Korea; 5 Department of Neurology, Bundang Clinical Neuroscience Institute, Seoul National University Bundang Hospital, Seongnam, Korea; 6 Department of Neurology, Kangnam Sacred Heart Hospital, Hallym University College of Medicine, 1 Singil-ro, Yeongdeungpo-gu, Seoul 07441 Korea

**Keywords:** Anxiety, Depression, Headache, Insomnia, Tension-type headache

## Abstract

**Background:**

Tension-type headache (TTH) represents the most common type of headache among the general population. Although such headaches are usually mild in severity, some individuals with TTH experience severe symptoms and psychiatric comorbidities. Such patients may also experience sleep disturbances, which have been associated with headache exacerbation. Nevertheless, information regarding the prevalence and impact of insomnia among individuals with TTH in a population-based setting is limited. Therefore, the aim of the present study was to evaluate the prevalence and impact of insomnia among individuals with TTH using data from the Korean Headache-Sleep Study (KHSS).

**Methods:**

We analysed data from the KHSS—a nation-wide, cross-sectional, population-based survey on headache and sleep involving Korean adults aged 19 to 69 years. Insomnia was defined as an Insomnia Severity Index score ≥ 10.

**Results:**

Among 2695 participants, 570 (21.2%) and 290 (10.8%) were classified as having TTH and insomnia, respectively. Among individuals with TTH, 113 (19.8%) met the criteria for probable migraine (PM). The prevalence of insomnia among individuals with TTH was significantly higher than that among individuals without headache (13.2% vs. 5.8%, *p < 0.001*). However, among the TTH group, the prevalence of insomnia did not significantly differ between participants fulfilling PM criteria and those not fulfilling PM criteria (14.2% vs. 12.9%, *p = 0.725*). Among individuals with TTH, headache frequency [median and interquartile range (IQR): 1.0 (0.3–3.0) vs. 0.4 (0.2–1.0), *p = 0.002*], visual analogue scale scores for headache intensity [median and IQR: 5.0 (4.0–7.0) vs. 4.0 (3.0–6.0), *p < 0.001*], Headache Impact Test-6 scores [median and IQR: 46.0 (40.0–52.0) vs. 42.0 (38.0–46.0), *p < 0.001*], anxiety prevalence (28.0% vs. 6.7%, *p <* 0.001), and depression prevalence (21.3% vs. 1.6%, *p < 0.001*) were significantly higher in those with insomnia than in those without insomnia.

**Conclusions:**

Our findings indicate that insomnia is prevalent among individuals with TTH. Moreover, insomnia was associated with exacerbation of headache symptoms and psychiatric comorbidities. Therefore, identification of insomnia among individuals with TTH is required to improve the management of headache symptoms in such patients.

**Electronic supplementary material:**

The online version of this article (10.1186/s10194-017-0805-3) contains supplementary material, which is available to authorized users.

## Background

Tension-type headache (TTH) is the most common form of primary headache among the general population, with a lifetime prevalence ranging from 20 to 87% [1]. TTH is usually associated with mild symptoms, and has been regarded as relatively non-severe since the condition is not life-threatening. However, some individuals with TTH experience frequent and severe headaches, which can decrease the ability to function at work, school, or home. Due to its high prevalence, the Global Campaign against Headache—a collaborative effort among the World Health Organization and three non-governmental organisations—has reported that the rate of disability due to TTH is greater than that due to migraine [1]. Therefore, the identification of factors associated with TTH represents an important public health issue.

Individuals who experience frequent headaches, including those with TTH, often report experiencing insomnia as well. Research has demonstrated that both lack of sleep and excessive sleep can trigger TTH [2, 3], and that sleep disturbances are associated with an increased risk of chronic tension-type headache (CTTH) among individuals with frequent episodic tension-type headaches (ETTH) [4].

Insomnia is a relatively common condition, affecting 10–30% of the general population [5]. Individuals with insomnia tend to exhibit limited functional capacity and decreased quality of life due to a variety of symptoms, including headaches [6, 7]. Cross-sectional studies have revealed that individuals with headaches exhibit increased odds ratios (ORs) for insomnia relative to those for individuals without headache [8, 9]. Additional research has indicated that the prevalence of insomnia is higher in patients with TTH than in individuals without headache [10]. Longitudinal studies have demonstrated that patients with migraine (OR =1.7) and non-migraineous headache (OR =1.4) are at increased risk for insomnia at the 11-year follow-up. Furthermore, individuals with insomnia exhibited an increased risk for TTH (relative risk [RR] = 1.4) and migraine (RR =1.4) at the 11-year follow-up [11, 12]. Several clinical studies have demonstrated an association between insomnia and exacerbation of TTH symptoms [13, 14]. Nevertheless, information regarding the impact of insomnia on the clinical presentation of TTH in a population-based setting is limited. We hypothesised that, among individuals with TTH, those with insomnia experience more severe symptoms than those without insomnia. Therefore, the objectives of the present study were as follows: 1) to evaluate the prevalence of insomnia and TTH in a general population-based sample, 2) to assess clinical characteristics and comorbidities of TTH according to the presence of insomnia, and 3) to investigate the association between TTH and insomnia including covariates such as sociodemographic factors, sleep time, sleep quality, and psychiatric comorbidities.

## Methods

### Survey

The present study utilised data from the Korean Headache-Sleep Study (KHSS), the design and methods of which have been described in detail in a previous report [15]. Briefly, a two-stage clustered random-sampling method was adopted for all Korean territories except Jeju-do, proportional to population distribution. The survey was performed via door-to-door visits and face-to-face interviews. The questionnaire utilised during these interviews exhibited sensitivity and specificity values of 86.2 and 75.5% for TTH diagnosis, respectively [16]. All interviewers were non-medical employees of Gallup Korea. The study protocol was approved by the Institutional Review Board and Ethics Committee of Hallym University Sacred Heart Hospital (Approval No. 2011-I077). Informed consent was obtained from each participant prior to each interview.

### Assessment of TTH

Diagnoses of TTH were based on criteria B to D for infrequent TTH (code 2.1) as listed in the third edition of the International Classification of Headache Disorders, beta version (ICHD-3 beta) (B: attack duration ranging from 30 min to 7 days; C: any two of four typical headache characteristics [i.e., bilateral location, non-pulsating quality, mild-to-moderate pain intensity, and no aggravation with movement]; D: attacks associated with both of the following: no nausea or vomiting and no more than either photophobia or phonophobia). Participants fulfilling all criteria were classified as having TTH. In accordance with ICHD-3 beta criteria, participants meeting criteria for both TTH and probable migraine (PM) were considered to have TTH.

### Assessment of insomnia

The self-reported Insomnia Severity Index (ISI) was used to evaluate the presence and severity of insomnia [17]. Participants with ISI scores of 10 or more were classified as having insomnia [18].

### Assessment of sleep time and sleep quality

Data regarding typical sleep times on workdays and free days during the previous month were collected and analysed. Average sleep time was defined as follows: [(workday sleep time × 5) + (free-day sleep time × 2)] / 7. Short sleep time was defined as an average sleep time ≤ 6 h per day. The Pittsburgh Sleep Quality Index (PSQI) was used to evaluate the quality of sleep in the present study. The PSQI is a self-administered questionnaire composed of 19 questions designed to assess seven components of perceived sleep status, including subjective sleep quality, sleep latency, sleep duration, habitual sleep insufficiency, sleep disturbance, use of sleep medications, and daytime dysfunction. Poor sleep quality was defined as a global PSQI score of 6 or more [19].

### Assessment of anxiety and depression

The Goldberg Anxiety Scale (GAS) was used to assess the prevalence of anxiety among participants. The GAS comprises four screening items and five supplementary items [20]. Participants who responded positively to two or more screening items and five or more total items were considered to have anxiety. The present study utilised the Korean version of the GAS, which has a sensitivity of 82.0% and a specificity of 94.4% for the diagnosis of anxiety [21]. The Korean version of the Patient Health Questionnaire-9 (PHQ-9), which has a sensitivity of 81.1% and specificity of 89.9% for the diagnosis of depression, was used to screen for depression in the present study [22, 23]. PHQ-9 scores of 10 or more were considered indicative of depression.

### Data analyses

The Kolmogorov–Smirnov test was used to evaluate the normality of the distribution. After confirming the normality of the distribution, Student’s t-tests and Chi-square tests were utilised to compare prevalence rates where appropriate. If the normality of the distribution was not confirmed, Mann-Whitney *U* tests were used to compare data between two groups. All statistical analyses were performed using Statistical Package for Social Sciences version 22.0 (SPSS 22.0; IBM, Armonk, NY, USA). The significance level was set at *p < 0.05* for all analyses.

Univariable and multivariable analyses were performed to determine factors contributing to insomnia among individuals with TTH. Among individuals with TTH, factors exhibiting significant differences between those with insomnia and those without insomnia were considered for univariable analyses. For multivariable analyses, three models were developed to examine the association between insomnia and TTH. Model 1—which included sociodemographic variables (age, sex, size of residential area, and educational level), anxiety, and depression—was used to investigate the association between insomnia and psychiatric conditions. Model 2—which included sociodemographic variables, short sleep time, and poor sleep quality as covariates—was used to investigate the association between insomnia and sleep-related parameters. Finally, Model 3—which included sociodemographic variables, anxiety, depression, short sleep time, and poor sleep quality as covariates—was used to investigate the association among insomnia, psychiatric conditions, and sleep-related parameters. Missing data occurred only with regard to educational level. All reported results are based on available data. Imputation techniques were not used to minimise non-response effects [24].

## Results

### Survey

**Fig. 1:**
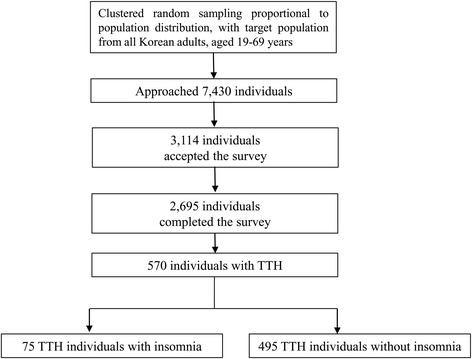
Patient flow chart in the Korean Headache-Sleep Study. *TTH:* Tension-type headache

Among 7430 individuals contacted by our interviewer, a total of 3114 provided consent for the survey, including the 2695 individuals who had completed the survey (cooperation rate: 36.2%, Fig. 1). No significant differences in the distributions of age, sex, size of residential area, or level of education were observed between the study population and the general population in Korea (Table 1).

**Table 1:** Sociodemographic characteristics of survey participants and total Korean population and cases identified as having TTH or insomnia

	Survey participants	Total population	P	Tension-type headache	*P*	Insomnia	*P*
	N (%)	N (%)		N, % (95% CI)		N, % (95% CI)	
Sex							
Men	1345 (49.3)	17,584,365 (50.6)	0.854^a^	268, 19.9 (17.8–22.0)	0.120^b^	117, 8.7 (7.2–1.2)	0.001^c^
Women	1350 (50.7)	17,198,350 (49.4)		302, 22.3 (20.1–24.6)		173, 12.4 (11.0–14.6)	
Age							
19–29	542 (20.5)	7,717,947 (22.2)	0.917^a^	119, 22.0 (18.5–25.5)	0.971^b^	59, 10.9 (8.3–13.5)	0.427^c^
30–39	604 (21.9)	8,349,487 (24.0)		127, 21.0(17.8–24.3)		53, 8.8 (6.5–11.0)	
40–49	611 (23.1)	8,613,110 (24.8)		131, 21.4 (18.2–24.7)		66, 10.8 (8.3–13.3)	
50–59	529 (18.9)	6,167,505 (17.7)		107, 20.2 (16.8–23.7)		63, 11.9 (9.1–14.7)	
60–69	409 (15.6)	3,934,666 (11.3)		86, 21.0 (17.1–25.0)		49, 12.0 (8.8–15.1)	
Size of residential area							
Large city	1248 (46.3)	16,776,771 (48.2)	0.921^a^	251, 20.1 (17.9–22.4)	0.004^b^	136, 10.9 (9.2–12.6)	0.943^c^
Medium-to-small city	1186 (44.0)	15,164,345 (43.6)		243, 20.5 (18.2–22.8)		125, 10.5 (8.8–12.2)	
Rural area	261 (9.7)	2,841,599 (8.2)		76, 29.1 (23.6–34.7)		29, 11.1 (7.3–14.9)	
Education level							
Middle school or less	393 (14.9)	6,608,716 (19.0)	0.752^a^	96, 24.5 (20.1–28.7)	0.327^b^	62, 15.8 (12.1–19.4)	0.006^c^
High school	1208 (44.5)	15,234,829 (43.8)		247, 20.5 (18.2–22.7)		116, 9.6 (7.9–11.3)	
College or more	1068 (39.6)	12,939,170 (37.2)		223, 20.9 (18.4–23.3)		109, 10.2 (8.4–12.0)	
Not responded	26 (1.0)			4, 15.4 (0.5–30.2)		3, 11.5 (0.0–24.7)	
Total	2695 (100.0)	34,782,715 (100.0)		570, 21.2 (19.6–22.7)		290, 10.8 (9.6–11.9)	

*N* number, *CI* confidence interval, *TTH* tension-type headache

^a^Comparison of sex, age group, size of residential area, and educational level distributions between the sample in the present study and the total population of Korea

^b^Comparison of sex, age group, size of residential area, and educational level distributions among survey participants

^c^Comparison of sex, age group, size of residential area, and educational level distributions among survey participants

### Prevalence of TTH, non-headache controls, and insomnia

Among the 2695 participants, 570 (21.2%) experienced TTH during the previous year, while 1422 (52.8%) did not. Among individuals with TTH, 113 (19.8%) also met the criteria for PM. The prevalence of TTH was not significantly affected by age, sex, or educational level. However, the prevalence of TTH was higher in rural areas than in large or small-to-medium cities (Table 1). A total of 290 (10.8%) participants were classified as having insomnia. Insomnia was more prevalent among women than men, and among those with lower levels of education (middle school or less) than those with higher levels of education. The prevalence of insomnia tended to increase with increasing age (Table 1). Sociodemographic characteristics of individuals with TTH and those without headache are summarised in Additional file 1: Table S1.

### Prevalence of anxiety, depression, short sleep time and poor sleep quality

A total of 268 (9.9%) participants exhibited symptoms of anxiety, while 116 (4.3%) exhibited symptoms of depression. The prevalence of anxiety (9.5% vs. 5.3%, *p = 0.001*) and depression (4.2% vs. 1.8%, *p = 0.001*) was significantly higher among individuals with TTH than among individuals without headache. A total of 469 (17.4%) participants reported an average sleep duration ≤6 h, and were thus classified as having short sleep time. Poor sleep quality was noted in 715 (26.5%) participants.

### Prevalence of insomnia among individuals with TTH

Among the 570 participants with TTH, 75 (13.2%) were classified as having insomnia. The prevalence of insomnia among participants with TTH was significantly higher than that among participants without headache (13.2% vs. 5.8%, *p < 0.001*).

**Fig. 2:**
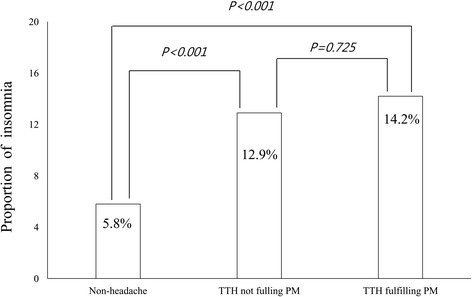
Prevalence of insomnia among individuals without headache, those with TTH fulfilling PM criteria, and those with TTH not fulfilling PM criteria. *PM,* probable igraine; *TTH,* tension-type headache

Previous studies have demonstrated a significant association between insomnia and migraine [12, 25]. Therefore, migrainous features may affect the prevalence of insomnia. We assessed the prevalence of insomnia among participants with TTH according to fulfillment of PM criteria. Among participants with TTH, the prevalence of insomnia did not significantly differ between those fulfilling PM criteria and those not fulfilling PM criteria (14.2% vs. 12.9%, *p = 0.725*). The prevalence of insomnia was significantly higher among participants with TTH not fulfilling PM criteria than among individuals without headache (12.9% vs. 5.8%, *p < 0.001*) (Fig. 2). We then analysed the prevalence of insomnia according to headache frequency. The prevalence of insomnia was significantly higher among participants with 1–10 TTH attacks per month than among those with < 1 TTH attack per month (17.1% vs. 10.4%, *p = 0.022*). However, the prevalence of insomnia did not significantly differ between participants with > 10 TTH attacks per month and those with < 1 TTH attack per month (14.3% vs. 10.4%, *p* = 0.575).

### Demographic characteristics and clinical presentation of TTH according to the presence of insomnia

Among individuals with TTH, those with insomnia exhibited significantly higher headache frequency per month, visual analogue scale (VAS) scores for headache intensity, and Headache Impact Test-6 scores than those without insomnia. Anxiety and depression were more prevalent among individuals with TTH and insomnia than among those without insomnia (Table 2).

**Table 2:** Demographics and clinical presentation of individuals with TTH according to the presence of insomnia

	TTH with insomnia, *N* = 75	TTH without insomnia, *N* = 495	*P*
Demographics			
Age, Mean ± SD (years)	44.5 ± 13.7	42.5 ± 13.7	0.236
Women, N (%)	43 (57.3)	259 (52.3)	0.418
Headache characteristics			
Bilateral pain, N (%)	45 (60.0)	329 (66.5)	0.272
Non-pulsating quality, N (%)	29 (38.7)	198 (40.0)	0.824
Mild-to-moderate severity, N (%)	73 (97.3)	490 (99.0)	0.225
Non-aggravation by movement, N(%)	58 (77.3)	393 (79.4)	0.682
Associated symptoms			
Photophobia, N (%)	6 (8.0)	40 (8.1)	0.981
Phonophobia, N (%)	31 (41.3)	150 (30.3)	0.056
Headache frequency, Median (IQR)	1.0 (0.3–3.0)	0.4 (0.2–1.0)	0.002^a^
VAS score, Median (IQR)	5.0 (4.0–7.0)	4.0 (3.0–6.0)	< 0.001^a^
HIT-6 score, Median (IQR)	46.0 (40.0–52.0)	42.0 (38.0–46.0)	< 0.001^a^
Anxiety, N (%)	21 (28.0)	33 (6.7)	< 0.001
Depression, N (%)	16 (21.3)	8 (1.6)	< 0.001

*N* number, *SD* standard deviation, *IQR* interquartile range

^a^Mann-Whitney U test

### Univariable and multivariable analyses for factors contributing to insomnia among individuals with TTH

Univariable analyses revealed that anxiety [OR = 5.4, 95% Confidence interval (CI): 2.9–10.0], depression (OR = 16.5, 95% CI: 6.8–40.2), short sleep time (OR = 3.1, 95% CI: 1.9–5.3), and poor sleep quality (OR = 12.8, 95% CI: 7.1–23.0) were associated with an increased risk of insomnia among individuals with TTH. Multivariate analyses including sociodemographic variables, anxiety and depression (Model 1) indicated that patients with anxiety (OR = 3.9, 95% CI: 2.0–8.0) and depression (OR = 14.8, 95% CI: 5.5–39.7) exhibited increased ORs for insomnia. In Model 2 (including sociodemographic variables, short sleep time, and poor sleep quality), poor sleep quality (OR = 13.4, 95% CI: 7.1–25.3) was significantly associated with insomnia status. In Model 3 (including sociodemographic variables, anxiety, depression, short sleep time, and poor sleep quality), anxiety (OR = 3.0, 95% CI: 1.4–6.7), depression (OR = 5.8, 95% CI: 2.0–16.3), and poor sleep quality (OR = 9.9, 95% CI: 5.1–19.2) were significantly associated with insomnia status (Table 3).

**Table 3:** Univariable and multivariable logistic regression analyses for contributing factors of insomnia among individuals with tension-type headache (*N* = 570)

	Univariable analysis	Multivariable analysis	Multivariable analysis	Multivariable analysis
	OR, 95% CI, *P-value*	Model 1	Model 2	Model 3
Sex				
	1.2 (0.8–2.0), *p = 0418*	1.2 (0.7–2.0), *p = 0.609*	1.3 (0.8–2.4), *p = 0.305*	1.2 (0.7–2.2), *p = 0.528*
Age				
20s	Reference			
30s	0.8 (0.3–1.8), *p = 0.556*	0.7 (0.2–1.6), *p = 0.360*	0.6 (0.2–1.5), *p = 0.256*	0.5 (0.2–1.4), *p = 0.220*
40s	1.1 (0.5–2.4), *p = 0.772*	1.1 (0.5–2.5), *p = 0.833*	1.1 (0.5–2.6), *p = 0.836*	1.1 (0.4–2.6), *p = 0.910*
50s	1.6 (0.8–3.4), *p = 0.206*	1.7 (0.7–4.2), *p = 0.246*	0.8 (0.3–2.1), *p = 0.704*	1.0 (0.4–2.8), *p = 0.931*
60s	1.3 (0.6–3.0), *p = 0.485*	1.2 (0.4–3.5), *p = 0.689*	0.9 (0.3–2.6), *p = 0.789*	0.9 (0.3–3.0), *p = 0.928*
Size of residential area				
Large city	Reference			
Medium-to-small city	1.2(0.7–2.0), *p = 0.508*	1.2 (0.7–2.1), *p = 0.566*	1.4(0.8–2.4), *p = 0.295*	1.3(0.7–2.4), *p = 0.395*
Rural area	0.5(0.2–1.2), *p = 0.125*	0.5 (0.2–1.5), *p = 0.244*	0.5(0.2–1.6), *p = 0.268*	0.6(0.2–1.8), *P = 0.340*
Education level				
Middle school or less	Reference			
High school	0.9(0.5–1.7), *p = 0.732*	0.9(0.4–2.0), *p = 0.716*	0.6(0.2–1.5), *p = 0.260*	0.6(0.2–1.4), *p = 0.215*
College or more	0.7(0.3–1.3), *p = 0.226*	0.8(0.3–2.1), *p = 0.622*	0.5(0.2–1.4), *p = 0.190*	0.5(0.2–1.6), *p = 0.250*
Not responded	1.8(0.2–18.5), *p = 0.621*	2.1(0.2–22.2), *p = 0.543*	1.9(0.1–28.8), *p = 0.626*	2.3(0.2–32.9), *p = 0.539*
Anxiety	5.4 (2.9–10.0), *p < 0.001*	3.9(2.0–8.0), *p < 0.001*		3.0 (1.4–6.7), *p = 0.006*
Depression	16.5 (6.8–40.2), *p < 0.001*	14.8(5.5–39.7), *p < 0.001*		5.8 (2.0–16.3), *p = 0.001*
Short sleep time	3.1 (1.9–5.3), *p < 0.001*		1.2(0.7–2.3), *p = 0.538*	1.2 (0.6–2.4), *p = 0.570*
Poor sleep quality	12.8 (7.1–23.0), *p < 0.001*		13.4(7.1–25.3), *p < 0.001*	9.9 (5.1–19.2), *p < 0.001*

Model 1 included sociodemographic variables (sex, age, size of residential area, level of education), anxiety, and depression

Model 2 included sociodemographic variables, short sleep time (≤6 h), and poor sleep quality (PSQI ≥ 6)

Model 3 included sociodemographic variables, anxiety, depression, short sleep time, and poor sleep quality

## Discussion

The main findings of the present study were as follows: 1) The prevalence of insomnia was significantly higher among individuals with TTH than among individuals without headache; 2) headache frequency, headache intensity, and impact of headache on functioning were significantly greater among participants with TTH and insomnia than among those without insomnia; and 3) anxiety, depression, and poor sleep quality were significant risk factors for insomnia among participants with TTH.

Previous studies have demonstrated a significant association between insomnia and TTH. For example, a community-based study in Hong Kong revealed that individuals with TTH exhibit increased ORs for insomnia relative to those for individuals without headache (OR = 2.2) [26]. A population-based study conducted in Norway further reported that the prevalence of insomnia is 1.8 times higher in individuals with TTH than in those without headache [10]. The findings of the present study are in agreement with the results of these previous studies, as our analyses indicated that the prevalence of insomnia was approximately two times higher among individuals with TTH than among those without headache. Therefore, our findings support the association between TTH and insomnia, and the similarity between our data and values reported in previous studies suggests that the methods of the present study were appropriate for evaluating this association.

One possible explanation for the observed association between insomnia and TTH involves the shared comorbidities of anxiety and depression. Indeed, anxiety and depression are common among patients with insomnia, and previous studies have indicated that both anxiety and depression are closely associated with the clinical presentation of insomnia [27]. Furthermore, insomnia symptoms tend to worsen as the severity of anxiety and/or depression [28]. Anxiety and depression are common among patients with headache [29, 30]. Although the prevalence of anxiety and depression among patients with TTH is lower than among patients with migraine, a significant proportion of individuals with TTH experience anxiety and depression [31]. In the present study, anxiety and depression were identified as significant risk factors for insomnia among individuals with TTH, even after adjusting for potential covariates. Alternatively, insomnia may trigger TTH [32]. Taken together, these findings indicate that the prevalence of TTH may be higher among individuals with insomnia than among those without insomnia. However, previous studies have reported that sleep deprivation and interruption of slow wave sleep can reduce pain threshold and cause hyperalgesia [33, 34]. Thus, insomnia may induce frequent headaches (including TTH) by decreasing the pain threshold.

Previous studies regarding the association between insomnia and TTH have not examined the impact of insomnia on the clinical presentation of TTH. In the present study, we observed that, among individuals with TTH, those with insomnia exhibited a higher frequency of headache and increased impact of headache on daily function than those without insomnia. In accordance with our findings, previous studies have indicated that individuals with a high frequency of TTH may experience increased disability, decreased quality of life, and a higher rate of comorbidities than those with low frequency of TTH [13]. Therefore, proper identification and management of insomnia may reduce the impact of headache among individuals with TTH. While pharmacological treatments for insomnia have demonstrated efficacy in randomised controlled trials, research has indicated that non-pharmacological alternatives such as cognitive behavioral therapy (CBT) are also effective [35].

In the present study, anxiety and depression were more common among individuals with TTH and insomnia than among those with TTH only (Table 2). Multivariable regression analyses revealed that anxiety and depression were significant contributing factors for insomnia among individuals with TTH (Table 3). Anxiety and depression often co-occur and have been closely associated with insomnia in previous studies [28, 36, 37]. Therefore, anxiety and depression may underlie the association between insomnia and TTH. However, further studies are required in order to clarify the associations among anxiety, depression, insomnia, and TTH.

Although there was no significant difference in the prevalence of insomnia between individuals with TTH fulfilling PM criteria and those not fulfilling PM criteria, the prevalence of insomnia among those fulfilling PM criteria was numerically higher than that among those not fulfilling PM criteria (Fig. 2). Considering that the prevalence of insomnia among individuals with migraine is much higher than that among those with TTH, our findings indicate that migrainous features may be substrates for the development of insomnia among individuals with TTH [9, 10, 25]. Further studies involving larger sample sizes are required to more fully elucidate the association between migrainous features and insomnia among individuals with TTH.

The present study possesses some limitations of note. First, polysomnography (PSG) was not performed to evaluate for sleep disorders such as sleep-disordered breathing. Some patients with sleep-disordered breathing present with symptoms of chronic insomnia [38]. Second, our study did not investigate the rate of chronic pain disorders such as fibromyalgia among participants. Since chronic pain disorders are also associated with psychiatric co-morbidities, future studies should investigate the association between chronic pain disorders and anxiety/depression in patients with headaches. Third, we did not evaluate the use of antidepressants, hypnotics, anxiolytics, or preventive medications for TTH, and were thus unable to examine the effects of such agents on TTH and insomnia. Fourth, although our study utilised a large sample size, the statistical power for executing some subgroup analyses was limited. That is, the lack of significant findings in certain subgroup analyses may have been due to the limited sample size.

Despite these limitations, our study possesses several strengths. First, the distributions of sex, age, size of residential area, and level of education for the study population reflected those observed in the general population, indicating that our findings may be applicable to the general population. Second, the characteristics of headache and psychiatric comorbidities were compared according to the presence of insomnia in patients with TTH. Third, we examined the association among TTH, insomnia, and closely related psychiatric comorbidities (i.e., anxiety and depression).

## Conclusion

Our findings indicate that insomnia is more prevalent among individuals with TTH than among those without headache. Moreover, among individuals with TTH, those with insomnia experienced more frequent headaches, increased rates of psychiatric comorbities, and more severe disability due to headache. Our findings suggest that insomnia represents an important comorbidity among individuals with TTH. Therefore, proper identification and management of insomnia may reduce the impact of headache among individuals with TTH.

## Additional file

Additional file 1: Table S1. Sociodemographic characteristics of participants with tension-type headache and non-headache controls. (DOCX 38 kb)

## Abbreviations

CBT: Cognitive behavioral therapy
CI: Confidence interval
CM: Chronic migraine
CTTH: Chronic tension-type headache
ETTH: Episodic tension-type headache
GAS: Goldberg Anxiety Scale
ICHD: International Classification of Headache Disorders
KHSS: Korean Headache-Sleep Study
OR: Odds ratio
PHQ-9: Patient Health Questionnaire-9
PM: Probable migraine
PSG: Polysomnography
PSQI: Pittsburgh Sleep Quality Index score
RR: Relative risk
TTH: Tension-type headache
VAS: Visual analogue scale

## References

[1] Stovner L, Hagen K, Jensen R, Katsarava Z, Lipton R, Scher A, Steiner T, Zwart JA (2007). The global burden of headache: a documentation of headache prevalence and disability worldwide. Cephalalgia.

[2] Spierings EL, Ranke AH, Honkoop PC (2001). Precipitating and aggravating factors of migraine versus tension-type headache. Headache.

[3] Rains JC, Davis RE, Smitherman TA (2015). Tension-type headache and sleep. Curr Neurol Neurosci Rep.

[4] Lyngberg AC, Rasmussen BK, Jorgensen T, Jensen R (2005). Prognosis of migraine and tension-type headache: a population-based follow-up study. Neurology.

[5] Morin CM, LeBlanc M, Daley M, Gregoire JP, Merette C (2006). Epidemiology of insomnia: prevalence, self-help treatments, consultations, and determinants of help-seeking behaviors. Sleep Med.

[6] Daley M, Morin CM, LeBlanc M, Gregoire JP, Savard J, Baillargeon L (2009). Insomnia and its relationship to health-care utilization, work absenteeism, productivity and accidents. Sleep Med.

[7] Bolge SC, Doan JF, Kannan H, Baran RW (2009). Association of insomnia with quality of life, work productivity, and activity impairment. Qual Life Res.

[8] Kim K, Uchiyama M, Liu X, Shibui K, Ohida T, Ogihara R, Okawa M (2001). Somatic and psychological complaints and their correlates with insomnia in the Japanese general population. Psychosom Med.

[9] Sutton DA, Moldofsky H, Badley EM (2001). Insomnia and health problems in Canadians. Sleep.

[10] Odegard SS, Engstrom M, Sand T, Stovner LJ, Zwart JA, Hagen K (2010). Associations between sleep disturbance and primary headaches: the third Nord-Trondelag health study. J Headache Pain.

[11] Odegard SS, Sand T, Engstrom M, Zwart JA, Hagen K (2013). The impact of headache and chronic musculoskeletal complaints on the risk of insomnia: longitudinal data from the Nord-Trondelag health study. J Headache Pain.

[12] Odegard SS, Sand T, Engstrom M, Stovner LJ, Zwart JA, Hagen K (2011). The long-term effect of insomnia on primary headaches: a prospective population-based cohort study (HUNT-2 and HUNT-3). Headache.

[13] Ong JC, Stepanski EJ, Gramling SE (2009). Pain coping strategies for tension-type headache: possible implications for insomnia?. J Clin Sleep Med.

[14] Sancisi E, Cevoli S, Vignatelli L, Nicodemo M, Pierangeli G, Zanigni S, Grimaldi D, Cortelli P, Montagna P (2010). Increased prevalence of sleep disorders in chronic headache: a case-control study. Headache.

[15] Cho SJ, Chung YK, Kim JM, Chu MK (2015). Migraine and restless legs syndrome are associated in adults under age fifty but not in adults over fifty: a population-based study. J Headache Pain.

[16] Kim B-K, Chu MK, Lee TG, Kim J-M, Chung C-S, Lee K-S (2012). Prevalence and impact of migraine and tension-type headache in Korea. J Clin Neurol.

[17] Bastien CH, Vallieres A, Morin CM (2001). Validation of the insomnia severity index as an outcome measure for insomnia research. Sleep Med.

[18] Morin CM, Belleville G, Belanger L, Ivers H (2011). The insomnia severity index: psychometric indicators to detect insomnia cases and evaluate treatment response. Sleep.

[19] Buysse DJ, Reynolds CF, Monk TH, Berman SR, Kupfer DJ (1989). The Pittsburgh sleep quality index: a new instrument for psychiatric practice and research. Psychiatry Res.

[20] Goldberg D, Bridges K, Duncan-Jones P, Grayson D (1988). Detecting anxiety and depression in general medical settings. BMJ.

[21] Lim JY, Lee SH, Cha YS, Park HS, Sunwoo S (2001). Reliability and validity of anxiety screening scale. J Korean Acad Fam Med.

[22] Pignone MP, Gaynes BN, Rushton JL, Burchell CM, Orleans CT, Mulrow CD, Lohr KN (2002). Screening for depression in adults: a summary of the evidence for the U.S. Preventive Services Task Force. Ann Intern Med.

[23] Choi HS, Choi JH, Park KH, Joo KJ, Ga H, Ko HJ, Kim SR (2007). Standardization of the Korean version of patient health questionnaire-9 as a screening instrument for major depressive disorder. J Korean Acad Fam Med.

[24] Little RJ, Rubin DB (2014) Statistical analysis with missing data. New York: John Wiley & Sons

[25] Lateef T, Swanson S, Cui L, Nelson K, Nakamura E, Merikangas K (2011). Headaches and sleep problems among adults in the United States: findings from the National Comorbidity Survey-Replication study. Cephalalgia.

[26] Yeung WF, Chung KF, Wong CY (2010). Relationship between insomnia and headache in community-based middle-aged Hong Kong Chinese women. J Headache Pain.

[27] Jansson-Frojmark M, Lindblom K (2008). A bidirectional relationship between anxiety and depression, and insomnia? A prospective study in the general population. J Psychosom Res.

[28] Johnson EO, Roth T, Breslau N (2006). The association of insomnia with anxiety disorders and depression: exploration of the direction of risk. J Psychiatr Res.

[29] Serafini G, Pompili M, Innamorati M, Gentile G, Borro M, Lamis DA, Lala N, Negro A, Simmaco M, Girardi P, Martelletti P (2012). Gene variants with suicidal risk in a sample of subjects with chronic migraine and affective temperamental dysregulation. Eur Rev Med Pharmacol Sci.

[30] Houle TT, Butschek RA, Turner DP, Smitherman TA, Rains JC, Penzien DB (2012). Stress and sleep duration predict headache severity in chronic headache sufferers. Pain.

[31] Song TJ, Cho SJ, Kim WJ, Yang KI, Yun CH, Chu MK (2016). Anxiety and depression in tension-type headache: a population-based study. PLoS One.

[32] Wang J, Huang Q, Li N, Tan G, Chen L, Zhou J (2013). Triggers of migraine and tension-type headache in China: a clinic-based survey. Eur J Neurol.

[33] Onen SH, Alloui A, Gross A, Eschallier A, Dubray C (2001). The effects of total sleep deprivation, selective sleep interruption and sleep recovery on pain tolerance thresholds in healthy subjects. J Sleep Res.

[34] Lentz MJ, Landis CA, Rothermel J, Shaver JL (1999). Effects of selective slow wave sleep disruption on musculoskeletal pain and fatigue in middle aged women. J Rheumatol.

[35] Geiger-Brown JM, Rogers VE, Liu W, Ludeman EM, Downton KD, Diaz-Abad M (2015). Cognitive behavioral therapy in persons with comorbid insomnia: a meta-analysis. Sleep Med Rev.

[36] Ohayon MM, Roth T (2003). Place of chronic insomnia in the course of depressive and anxiety disorders. J Psychiatr Res.

[37] Oh K, Cho SJ, Chung YK, Kim JM, Chu MK (2016). Erratum to: combination of anxiety and depression is associated with an increased headache frequency in migraineurs: a population-based study. BMC Neurol.

[38] American Academy of Sleep Medicine (2014) International classification of sleep Sisorders—third edition (ICSD-3). American Academy of Sleep Medicine Resource Library Avialble via DIALOG. http://www.aasmnet.org/library/default.aspx. Accessed 20 Aug 2017.

