# Supplementary material for: Insomnia in tension-type headache: a population-based study
Source: J Headache Pain. 2017 Sep 12;18(1):95. doi: 10.1186/s10194-017-0805-3 (PMC5595708; doi:10.1186/s10194-017-0805-3)
Supplement: Additional file 1: Table S1. — Sociodemographic characteristics of participants with tension-type headache and non-headache controls. (DOCX 38 kb) [file 10194_2017_805_MOESM1_ESM.docx]

|  | | All survey participants, N | Tension-type headache, N (%) | Non-headache controls, N (%) | P value^a^ |
| --- | --- | --- | --- | --- | --- |
| Gender | |  |  |  | *< 0.001* |
|  | Male | 1345 | 268 (19.9) | 838 (62.3) |  |
|  | Female | 1350 | 302 (22.3) | 584 (43.3) |  |
| Age | |  |  |  | *0.405* |
|  | 19–29 | 542 | 119 (22.0) | 286 (52.8) |  |
|  | 30–39 | 604 | 127 (21.0) | 293 (48.5) |  |
|  | 40–49 | 611 | 131 (21.4) | 295 (48.3) |  |
|  | 50–59 | 529 | 107 (20.2) | 303 (57.3) |  |
|  | 60–69 | 409 | 86 (21.0) | 245 (59.9) |  |
| Size of the residential area | |  |  |  | *0.009* |
|  | Large city | 1248 | 251 (20.1) | 647 (51.8) |  |
|  | Medium-to-small city | 1186 | 243 (20.5) | 650 (54.8) |  |
|  | Rural area | 261 | 76 (29.1) | 125 (47.9) |  |
| Education level | |  |  |  | *0.345* |
|  | Middle school or less | 393 | 96 (24.5) | 208 (52.4) |  |
|  | High school | 1208 | 247 (20.5) | 646 (53.5) |  |
|  | College or more | 1068 | 223 (20.9) | 551 (51.6) |  |
|  | Not responded | 26 | 4 (15.4) | 19 (73.1) |  |
| Total | | 2695 | 570 (21.2) | 1422 (52.8) |  |

Table S1. Sociodemographic characteristics of participants with tension-type headache and non-headache controls

^a^ Comparing between tension -type headache and non-headache controls
